# Identification of a Prognostic Model Based on 2-Gene Signature and Analysis of Corresponding Tumor Microenvironment in Alcohol-Related Hepatocellular Carcinoma

**DOI:** 10.3389/fonc.2021.719355

**Published:** 2021-09-27

**Authors:** Yong Guo, Jiejun Hu, Zhibo Zhao, Guochao Zhong, Jianping Gong, Dong Cai

**Affiliations:** ^1^ Department of Hepatobiliary Surgery, People’s Hospital of Changshou, Chongqing, China; ^2^ Department of Hepatobiliary Surgery, The Second Affiliated Hospital of Chongqing Medical University, Chongqing, China

**Keywords:** alcohol-related HCC, bioinformatics analysis, prognostic model, tumor microenvironment, immune cells

## Abstract

Hepatocellular carcinoma (HCC) is one of the most prevalent malignant tumors with the poor prognosis. Nowadays, alcohol is becoming a leading risk factor of HCC in many countries. In our study, we obtained the DEGs in alcohol-related HCC through two databases (TCGA and GEO). Subsequently, we performed enrichment analyses (GO and KEGG), constructed the PPI network and screened the 53 hub genes by Cytoscape. Two genes (BUB1B and CENPF) from hub genes was screened by LASSO and Cox regression analyses to construct the prognostic model. Then, we found that the high risk group had the worse prognosis and verified the clinical value of the risk score in alcohol-related HCC. Finally, we analyzed the tumor microenvironment between high and low risk groups through CIBERSORT and ESTIMATE. In summary, we constructed the two-gene prognostic model that could predict the poor prognosis in patients with alcohol-related HCC.

## Introduction

Liver cancer is the sixth most common malignancy and the third leading cause of cancer-related death worldwide by 2020 and hepatocellular carcinoma (HCC) is the most common type of liver cancer, accounting for 75%–85% cases (1). The common driving factors of HCC are viral hepatitis (HBV or HCV), fatty liver disease, diabetes, alcohol, aflatoxin and aristolochic acid (2). With the improvement of anti-virus therapies and increase of the alcohol consumption in many regions, alcohol may become a leading role of HCC in the future (3–5). Early retrospective studies indicated that the tumor stage at diagnosis was influenced by the etiology and alcohol-related HCC was diagnosed at a later stage (4, 6). Similarly, a prospective study found that patients with alcohol-related HCC have reduced overall survival time compared with patients with non–alcohol-related HCC and patients with alcohol-related HCC have worse liver function and tumor characteristics at diagnosis (7). Thus, early detection and diagnosis is crucial for the treatment and prognosis of alcohol-related HCC. Unfortunately, there are no effective markers for its detection and prognostic prediction.

Considering the development of sequencing technology and the reduction of costs, technologies of the gene sequencing and bioinformatic analysis have been widely used to screen potential biomarkers at the mRNA level and helped us identify the differentially expressed genes (DEGs) and functional pathways involved in the progression of cancer. Recent years, research combined with big data analysis is becoming a trend of future research in tumor. In addition, the tumor microenvironment (TME) included immune cells, stromal cells, endothelial cells, inflammatory cells, and fibroblasts (8), and increasing studies have showed that the compositions of TME can influence the treatment and prognosis of tumor (9–11). Therefore, in order to effectively improve the prognosis and treatment of tumor, it is necessary to understand the cell compositions and function of the TME.

In the current study, we screened the 2-gene signature from TCGA and GEO databases and constructed a prognostic model in alcohol-related HCC. Furthermore, we explored the TME between high and low risk groups in alcohol-related HCC based on the CIBERSORT and ESTIMATE algorithm.

## Materials And Methods

### Data Collection and Processing

We obtained the Gene Expression Quantification data(HTSeq-Counts) of RNA-Seq and corresponding clinical information of 377 HCC patients from the TCGA-LIHC cohort (https://portal.gdc.cancer.gov/) through the ‘TCGAbiolinks’ package that can access the National Cancer Institute (NCI) Genomic Data Commons (GDC) through the GDC Application Programming Interface (API). Next, we eliminated information of patients that didn’t meet the criteria. The exclusion criteria were as follows (1): alcohol consumption is not the only risk factor for HCC (2); incomplete clinical information(including prognostic, TNM staging, gender, age and risk factors information). Finally, we got the mRNA expression profile and corresponding clinical information of 68 patients with alcohol-related HCC from TCGA. For GEO data, we chose the mRNA pression profile of GSE59259 which contained 8 alcohol-related HCC tissues and paired 8 the cancer-free surrounding liver tissues as of May 05, 2015.

### Differentially Expressed Gene Screening

For TCGA data, we used the ‘DESeq2’ package in R software to obtain differentially expressed genes (DEGs) in alcohol-related HCC. Then DEGs with absolute log2 foldchange (FC) ≥2 and adjusted P value <0.05 were considered to be included for subsequent analysis. For GEO data, we used the ‘GEO2R’ to obtain DEGs in alcohol-related HCC and chose DEGs with absolute log2 foldchange (FC) ≥1 and adjusted P value <0.05 for subsequent analysis. Finally, we obtained the final DEGs through intersection of TCGA and GEO databases (http://bioinformatics.psb.ugent.be/webtools/Venn/).

### Functional Enrichment Analysis and PPI (Protein-Protein Interactions) Network Construction

Enrichment analysis of Gene Ontology (GO) and Kyoto Encyclopedia of Genes and Genomes (KEGG) pathway for final DEGs or hub genes was performed using the DAVID v6.8(https://david.ncifcrf.gov/tools.jsp) (12, 13).

We employed the STRING(v11.0) database with validated and conjectural PPI to obtain the corresponding PPI network. Subsequently, the MCODE (14) clustering algorithm was used for subnets screening. With the default settings, we chose the highest score subnet. Then, we employed the cytoHubba plugin (15) to calculate the degrees of genes in the subnet and genes with no less than 100 degrees were identified as hub genes. The above results were visualized by the ‘ggplot2’ package in R software or Cytoscape v3.7.

### Establishment of a Prognostic Signature Model and Survival Analysis

The least absolute shrinkage and selection operator(LASSO) and multivariate Cox regression analyses were used to study the correlation between the prognosis and gene expression levels. Firstly, we used LASSO regression analysis to identify genes associated with the prognosis in hub genes through R software. Secondly, we applied multivariate Cox regression to further narrow the range of alcohol-related HCC marker genes through SPSS v20 and finally obtained 2 genes as marker. A multi-gene prognostic risk score was established based on a combination of regression coefficients from the multivariate Cox regression model (β) multiplied by their mRNA expression levels. Risk score(RS) = (expression level of BUB1B * β) + (expression level of CENPF * β). Taking the median risk score as a cutoff value, 68 alcohol-related HCC patients from TCGA were divided into high(n=34) and low(n=34) risk groups. Kaplan–Meier survival curves and time-dependent receiver operational feature (ROC) curve analyses were made to assess the predictive capacity of the model.

### Validation of Independent Predictor Factors for Overall Survival

We used univariate and multivariate Cox analysis to study the independent prognostic value of the RS and other clinical characteristics, and further explored the independent prognostic value of the RS in different subgroups by stratified analysis.

### Estimating the Composition of Immune Cells

CIBERSORT is a deconvolution algorithm based on the principle of linear support vector regression used to describe the infiltration of immune cells in the sample (16). LM22 is composed of 547 genes that accurately distinguish 22 human hematopoietic cell phenotypes, including seven T cell types, naïve and memory B cells, plasma cells, NK cells, and myeloid subsets (16). We used CIBERSORT and LM22 to jointly estimate the scores of 22 human immune cell types in alcohol-related HCC specimens from the TCGA cohort. For each specimen, the sum of all estimated immune cell type scores was equal to 1. Then, we described the total distribution of all estimated immune cell types and compared differences in the composition of immune cell types between high and low risk groups. Furthermore, we discussed the differences of compositions of each immune cell type between high and low risk groups and analyzed the correlation between immune cell types (*P* < 0.05) and RS in different clinical characteristics subgroups.

## Results

### Study Process and Summary Of Patients’ Information in TCGA

**Figure 1:**
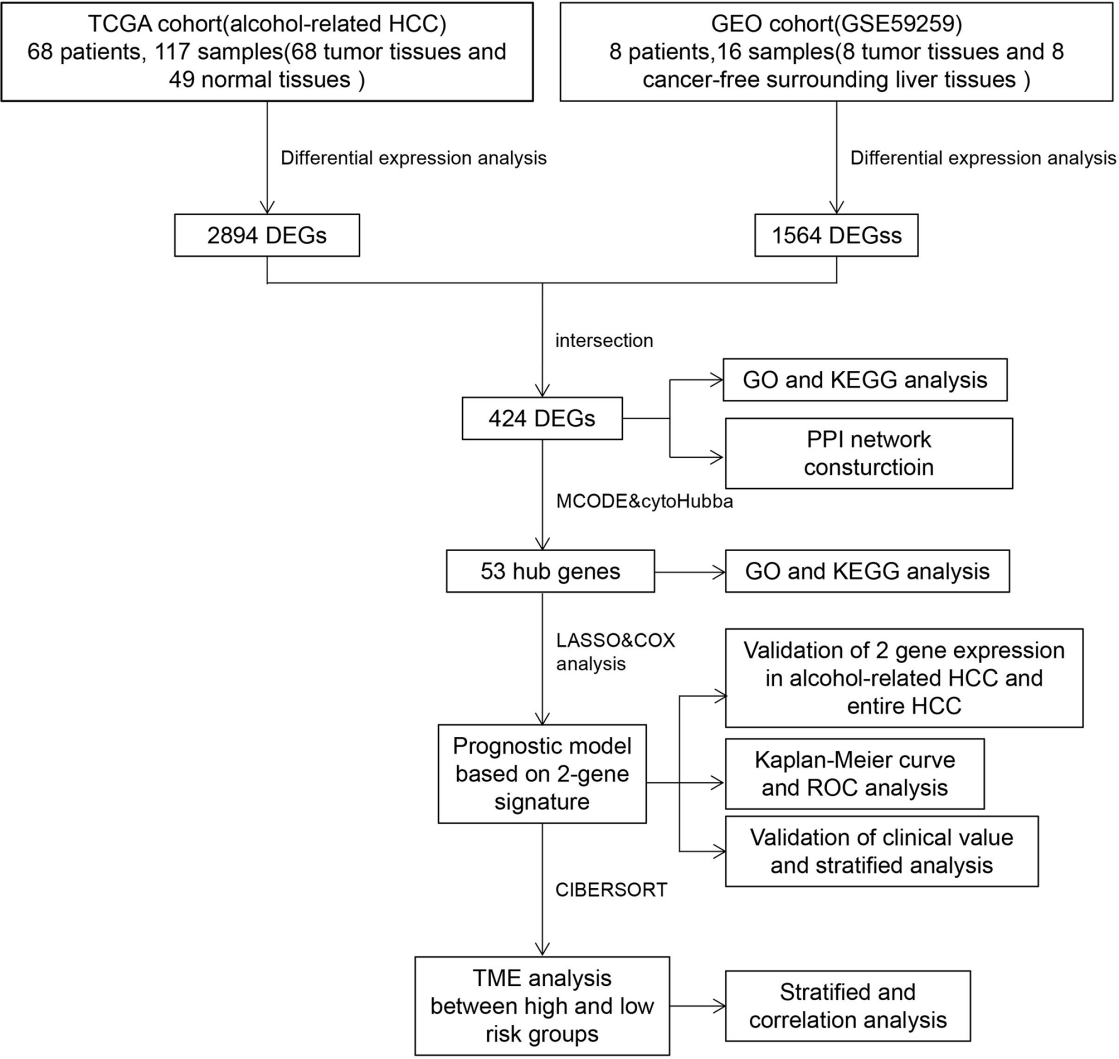
Overall flowchart of the study.

To better describe the entire process of our study, we developed a flowchart in **Figure 1**. We obtained clinical information of 377 patients from TCGA and excluded patients who were inconsistent with the purpose of our study or whose clinical data were missing. Finally, information of 68 alcohol-related HCC patients were obtained and presented in **Table 1**.

**Table 1:** Clinical information of alcohol-related HCC from TCGA.

Clinical characteristics		Total	%
TCGA (alcohol-related HCC)		68	100
Survival status	Survival	55	80.9
	Death	13	19.1
Age	≥60 years	41	60.3
	<60 years	27	39.7
Gender	Male	54	79.4
	Female	14	20.6
Stage	I	30	44.1
	II	16	23.5
	III	21	30.9
	IV	1	1.5

### Screening DEGs and Enrichment Analysis of DEGs

**Figure 2:**
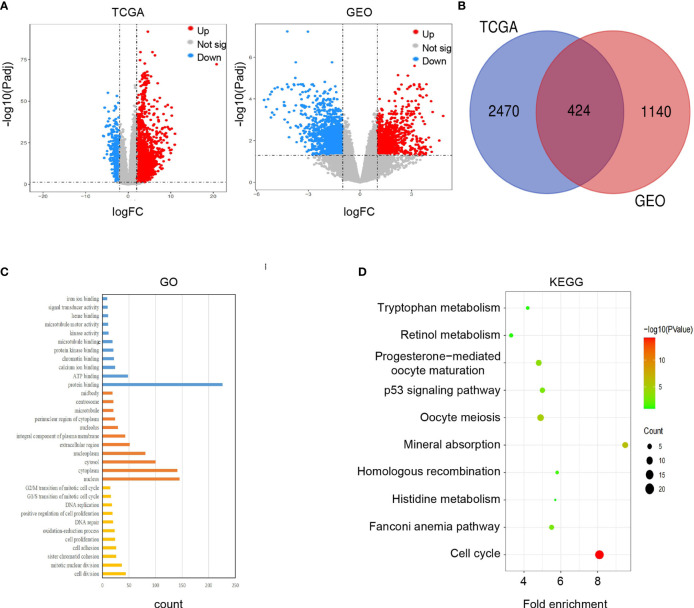
Screening and enrichment analysis of DEGs. **(A)** Volcano plots showed differentially expressed genes (DEGs) in alcohol-related HCC from TCGA and GEO. **(B)** Venn diagram showed final DEGs that are differentially expressed in both databases (TCGA and GEO). **(C)** GO analysis of DEGs. **(D)** KEGG analysis of DEGs.

Firstly, we completed a comparative analysis of mRNA expression profiles between alcohol-related HCC tissues(n=68) and normal tissues (n=49) and screened 2894 DEGs (| logFC | ≥ 2, adjusted *P* < 0.05) by ‘DESeq2’ algorithms. Similarly, we obtained 1564 DEGs(| logFC | ≥ 1, adjusted *P* < 0.05) between alcohol-related HCC tissues(n=8) and the cancer-free surrounding liver tissues(n=8) from GEO(GSE59259) by GEO2R (**Figure 2A**). Then, we intersected DEGs of TCGA and GEO, and finally obtained 424 DEGs for subsequent enrichment analysis (**Figure 2B**). Biological process(BP) and molecular function (MF) of GO analysis showed that these DEGs is mainly enriched in the cell division, mitotic nuclear division, sister chromatid cohesion and protein binding, ATP binding, calcium ion binding,respectively. More details about GO analysis of DEGs can be learned in **Figure 2C**. And KEGG analysis showed the key pathways correlated with the alcohol-related HCC samples: cell cycle, oocyte meiosis, mineral absorption, progesterone-mediated oocyte maturation, p53 signaling pathway, fanconi anemia pathway and Homologous recombination(*P* < 0.05)(**Figure 2D**).

### Identification of Hub Genes and Corresponding Enrichment Analysis

**Figure 3:**
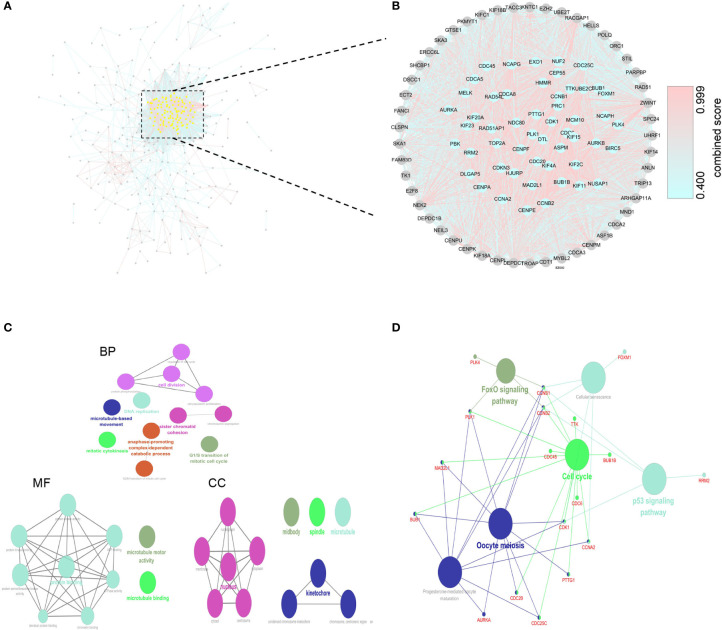
Construction of PPI network and corresponding enrichment analysis of hub genes. **(A)** PPI network of 424 DEGs. **(B)** The crucial module identified by MCODE. The grey or yellow dots represent genes or hub genes, respectively, and the color gradients from sky blue to rose hermosa represent the combined score of two genes. **(C)** GO analysis of hub genes. **(D)** KEGG analysis of hub genes.

We used 424 overlapping DEGs to construct a PPI network from STRING database (medium confidence ≥ 0.4). The network had 410 nodes and 5969 edges, with an average node degree of 29.1. The grey or yellow dots represent genes or hub genes, respectively, and the color gradients from sky blue to rose hermosa represent the combined score of two genes (**Figure 3A**). Then, we visualized the PPI network through Cytoscape 3.7. The core subnets were isolated *via* the MCODE plugin with the criteria of K-core ≥ 2, node score cut off = 0.2, degree cut-off = 2, and max depth = 100. We chose the highest score subnet (101 nodes and 4446 edges) and further selected 53 genes with degree ≥ 100 as hub genes through cytoHubba plugin (**Figure 3B**). Likewise, we performed GO and KEGG analyses of hub genes and visualized the results by Cytoscape 3.7 to find the key pathway. BP of GO analyses indicated that these genes mainly participated in the cell division, DNA replication, sister chromatid cohesion, anaphase-promoting complex-dependent catabolic process and G1/S transition of mitotic cell cycle. MF of GO analyses also indicated that these genes mainly participated in protein binding(including ATPase activity, ATP binding, protein kinase activity or binding, etc.), microtubule motor activity and microtubule binding (**Figure 3C**). Furthermore, KEGG analyses showed that these genes were mainly involved in cell cycle, oocyte meiosis, p53 signaling pathway, FoxO signaling pathway, cellular senescence and progesterone-mediated oocyte maturation (**Figure 3D**).

### Construction and Evaluation of 2-Gene Signature Prognostic Model

**Figure 4:**
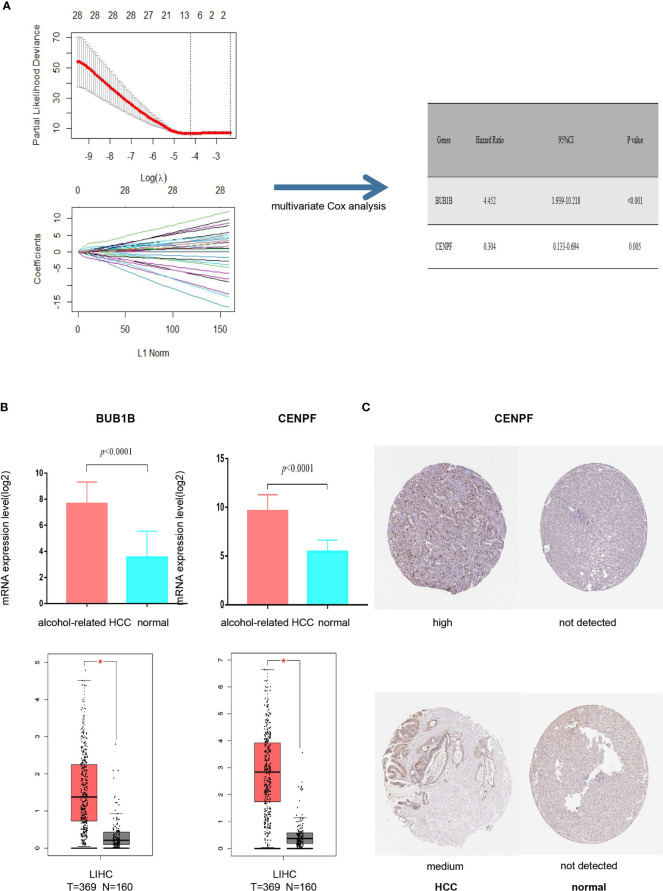
Screening prognostic genes and expression of the genes in alcohol-related HCC and entire HCC. **(A)** Process of screening prognostic genes. **(B)** The mRNA expression levels of two genes in alcohol-related HCC and entire HCC. **(C)** Protein expression level of CENPF in HCC. *P < 0.05.

It was found that deletion of the hub protein is more likely to be lethal than deletion of the non-hub protein (also known as the centrality-lethality rule), and this small fraction of genes is vital because the genes are linked to the survival of an organism (17). Thus, we firstly applied the LASSO regression analysis to identify 8 initial markers(CCNA2, BUB1B, BIRC5, AURKA, CENPF, KIF15, CEP55, PLK4) from 53 hub genes. In order to make the results more reliable, we further narrowed the range through the multivariate Cox regression analysis and finally obtained two genes (BUB1B and CENPF). The entire process of extracting stable markers from 53 hub genes in alcohol-related HCC to build a survival prediction model is presented in **Figure 4A**. Interestingly, we found the mRNA expression levels of both genes were higher in alcohol-related HCC specimens than in the normal specimens in TCGA cohort (**Figure 4B**). Consistently, the mRNA expression levels of both genes were higher in HCC specimens than in normal specimens by GEPIA(Gene Expression Profiling Interactive Analysis) that match TCGA normal and GTEx data (**Figure 4B**). We further explored the protein expression levels of the 2 genes in HPA(Human Protein Atlas) and found CENPF was more strongly stained in HCC specimens (**Figure 4C**) while BUB1B did not have sufficient information on IHC staining of HCC in HPA.

**Figure 5:**
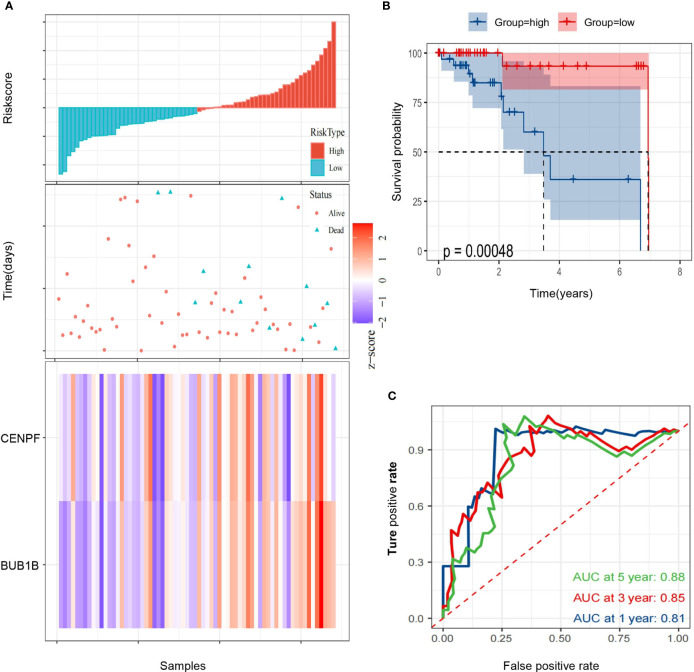
Identification of a 2-gene signature model associated with the overall survival of patients with alcohol-related HCC. **(A)** The risk scores distribution, survival status, and gene expression patterns of patients in high and low risk groups. **(B)** The Kaplan-Meier curve analysis of overall survival of alcohol-related HCC patients between high and low risk groups. **(C)** The time-dependent ROC curves analyses.

Then, we calculated the risk score of each HCC patient from TCGA and constructed a prognostic model based on two genes. RS(risk score) = BUB1B x 1.493 + CENPF x (-1.192). Subsequently, 68 HCC patients with follow-up information were divided into low risk group and high risk group according to the median value of risk scores in the TCGA (**Figure 5A**). The Kaplan–Meier survival curve was applied to demonstrate that patients in the high risk group had poorer outcomes than patients at the low risk group (**Figure 5B**). Besides, the time-dependent ROC curve was used to assess the prognostic ability of the two-gene signature, and AUCs of the 2-gene signature at 1, 3, and 5 years were 0.81, 0.85 and 0.88, respectively (**Figure 5C**).

### Stratified Analysis Based on Clinicopathological Features

**Figure 6:**
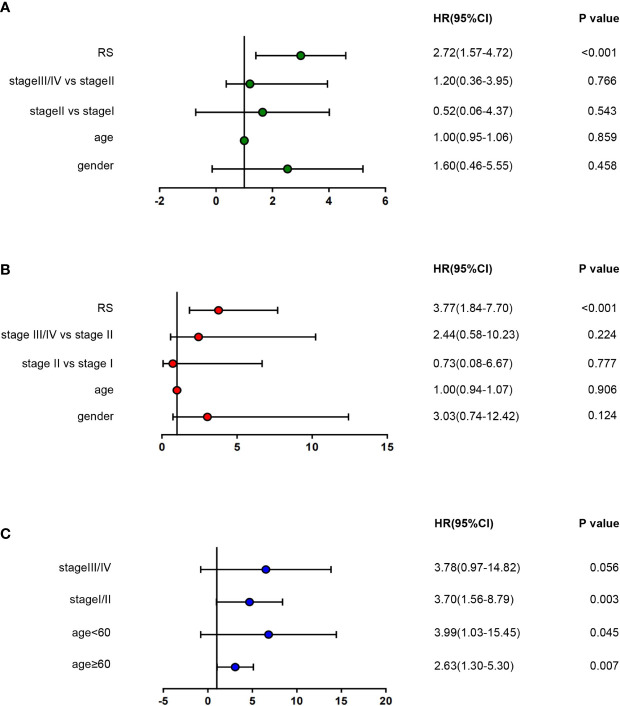
Cox regression analyses of the association between clinicopathological factors and overall survival (OS). **(A, B)** Univariate/multivariate Cox regression analyses of clinicopathological factors(including RS) and OS of patients in TCGA. **(C)** Univariate Cox regression analyses of RS and OS indifferent clinicopathological subgroups.

To assess whether our risk score could be more instructive than other clinicopathological features (age, gender, TNM staging), we used univariate and multivariate Cox regression analyses to assess independent predictive values for the two-gene signature in alcohol-related HCC patients. The hazard ratio of RS in univariate and multivariate Cox regression analyses is 2.72(1.57-4.72, *P <*0.001) and 3.77 (1.84-7.70, *P <*0.001), respectively. However, other clinicopathological features were not statistically significant in univariate and multivariate Cox regression (**Figures 6A, B**). Furthermore, we carried out a stratified analysis to illuminate the association between RS and survival in different clinicopathological features subgroups by univariate Cox regression analyses. We found that HR of RS in age <60, age ≥60 and stage I-II groups were 3.99(1.03-15.45, *P* =0.045), 2.63(1.30-5.30, *P* =0.007) and 3.70(1.56-8.79, *P* =0.003), respectively, while the HR of RS in stage III-IV group was not statistically significant (**Figure 6C**). These results suggest that RS can be a good independent prognostic factor.

### Analysis of Tumor Microenvironment Between High and Low Risk Groups

**Figure 7:**
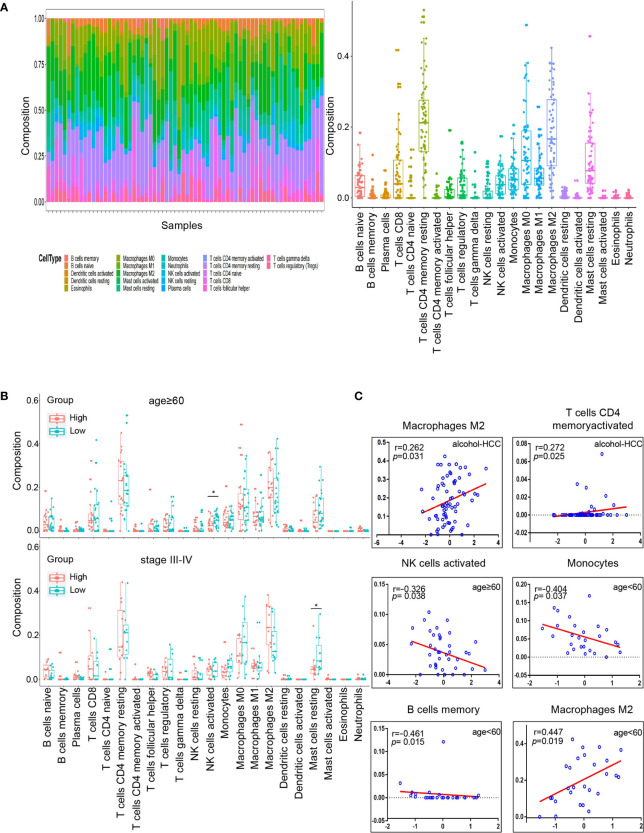
Tumor microenvironment analysis in high and low risk groups. **(A)** Relative proportion of 22 immune cells infiltrating in alcohol-related HCC patients. **(B)** Differences in 22 immune cells between the high and low groups in age subgroups. The top graph is the group that age≥60 and the bottom graph is the group that TNM staging III/IV. **(C)** Correlation analysis of immune cells and RS in different subgroups. The groups are entire groups of alcohol-related HCC, age ≥60 and <60 groups from left to right and from top to bottom. *P < 0.05.

We estimated the immune cell composition of 70 samples in alcohol-related HCC and quantified the relative levels of different cell types in the mixed cell population through CIBERSORT (**Figure 7A**). We compared different cell types of patients while there were no significant differences in all cell type comparisons between the low risk group and the high risk group (**Figure S1A**). Then, we further compared different cell types in different clinicopathological features subgroups. We found the composition of NK cells activated in the low risk group was higher (*P*=0.018) than in the high risk group among people aged ≥60. Similarly, we found that the composition of resting mast cells in the low risk group was higher (*P*=0.047) than in the high risk group among people with TNM staging III/IV (**Figure 7B**). Unfortunately, there was no significant difference in all cell type comparisons between the low risk group and the high risk group in age<60 or TNM staging I/II subgroups (**Figure S1B**). Similarly, we calculated the stromal and immune scores between the high and low risk groups through ESTIMATE. Unfortunately, there was no significantly difference in stromal or immune scores between the high and low risk groups (**Figures S2A, B**).

Subsequently, we analyzed the correlation between RS and compositions of all cell types across the whole or various subgroups. The results indicated that the compositions of T cells CD4 memory activated and macrophages M2 positively correlated (*P*<0.05) with RS in alcohol-related HCC. Furthermore, the composition of NK cells activated was negatively correlated (*P*<0.05) with RS in the age ≥60 group, which was consistent with the results of previous comparison. The compositions of B cells memory and monocytes were negatively correlated (*P*<0.05) with RS and the compositions of M2 macrophages was positively correlated (*P*<0.05) with RS in the age<60 group (**Figure 7C**). However, there was no correlation between the composition of all cell types and RS in the TNM staging groups. These results suggest that RS as an independent prognostic factor may be due to differences of immune cells in the tumor microenvironment among people with alcohol-related HCC.

Finally, we explored the correlation between the expression of the 2 genes and 22 immune cells through the Spearman correlation analysis. The results indicated the expression of CENPF was positively correlated (*P*<0.05) with the composition of B cells memory and negatively correlated (*P*<0.05) with the compositions of B cells naïve and monocytes. And the expression of BUB1B was positively correlated (*P*<0.05) with the compositions of T cells CD4 memory activated and T cells follicular helper (**Figure S2C**).

## Discussion

So far, HCC is still one of the most life-threatening malignancies in the world due to the complicated molecular mechanisms and microenvironment (18). Encouragingly, many treatments for HCC have been improved with the development of next-generation sequencing technology and targeted therapies (19). However, there are not always satisfactory for HCC targeted therapies in partial patients due to the differences of their clinicopathological features and genes (20). For example, it is well known that alcohol consumption is a risk factor for HCC and other cancers (21–25), but there are still many unknown molecular mechanisms and prognostic biomarkers in alcohol-related HCC. Therefore, prognostic biomarkers with higher prediction accuracy in predicting prognosis are urgently needed before detectable clinicopathological abnormalities in treatments of alcohol-related HCC patients.

In our study, We applied two different datasets to eliminate heterogeneity. Notedly, we found that the enrichment analysis results of DEGs and hub genes, including the cell cycle and p53 pathways, have been widely documented as the vital roles in HCC development (26, 27). Combined with the results of the prognostic model, our study suggested that BUB1B and CENPF may participate in the development of alcohol-related HCC.

In fact, several studies have showed that BUB1B promote tumor growth and metastasis in many solid tumors (28–30). Jiannan Qiu et al. found that the BUB1B, overexpressed in HCC, could inhibit apoptosis and prevent G0/G1 cell cycle arrest through activation of mTORC1 signaling pathway (30). CENPF, centromere protein F, is a transient kinetochore protein that regulates multiple cellular processes, including chromosome segregation during mitosis (31, 32). It has been documented that CENPF can interact with several key cell cycle checkpoint proteins and late telophase proteins, including syntaxin 4 and synaptosomal−associated protein 25, to further affect cellular processes (33, 34). Tang Hexiao et al. found that the knockdown of CENPF inhibited the progression of lung adenocarcinoma mediated by ERβ2/5 pathway (35). Similarly, the overexpression of CENPF has been observed in HCC tissues and the CENPF regulated by lymphoid‐specific helicase (LSH) can promote the growth of HCC (36). However, the specific molecular mechanisms of the two genes in alcohol-related HCC remain unclear. We hypothesized that the two genes may influence the cycle and metabolism of hepatocytes in the context of alcohol consumption.

Previous studies have shown that the TME plays a vital role in the occurrence and progression of cancer (37–39). Thus, we estimated the scores of 22 human immune cell types in alcohol-related HCC samples by CIBERSORT, and compared differences in the composition of immune cell types and the between high and low risk groups. Moreover, we analyzed the correlation between RS and compositions of all cell types in the whole or various subgroups. Interestingly, our results indicated that the carcinogenic effects of memory activated CD4 T cells and M2 macrophages may be further amplified in alcohol-related HCC (40, 41). Notedly, the compositions of the activated NK cells and resting mask cells have significant differences in subgroups of age ≥60 or TNM III/IV stages, respectively. This may be related to the fact that alcohol-related HCC is more easily detected in patients with poorer pathological status (7). Furthermore, our study indicated that M2 macrophages, CD4+ T cells, NK cells, B cells and monocytes may participated in the development of alcohol-related HCC, which may involve the activation of STAT3 and NF-kB transcription factors or the accumulation of ROS and iron (42). In the future research, we need explore the exact molecular mechanisms of these immune cells in alcohol-related HCC and learned whether CENPF and BUB1B can affect the development of alcohol-related HCC through the regulation of immune cells.

The most significant advantage of the present work is construction of the prognostic model in alcohol-related HCC. In fact, Xiuzhi Zhang et al. identified several key genes for alcohol-related hepatocellular carcinoma through bioinformatic analysis (43). Unfortunately, their work only screened out genes in alcohol-related HCC without establishing and analyzing the relevant prognostic model. Thus, we screened out the 2-gene signature and constructed a risk model to predict the prognosis in alcohol-related HCC. In addition, there is a limitation that we need to acknowledge in our study. There are only 68 patients with alcohol-related HCC from TCGA in our study, which results in a lack of external datasets to verify the stability of our model. Similarly, we did not perform the subgroup analysis of the gender due to the severe imbalance in the ratio of males to females.

In summary, our results indicated that a two-gene signature prognostic model could be used to predict the prognosis in alcohol-related HCC patients. Hopefully, the prognostic model could be a clinically beneficial tool for individualized treatment in patients with alcohol-related HCC.

## Data Availability Statement

The original contributions presented in the study are included in the article/**Supplementary Material**. Further inquiries can be directed to the corresponding author.

## Author Contributions

YG collected the data, analyzed and interpreted the data, and drafted the manuscript. JH interpreted the data and contributed to the substantial revisions of the manuscript. ZZ, GZ and JG helped to perform the statistical analysis and interpret the data. DC made contribution to the conception and design, analyzed and interpreted the data, supervised the study, revised the manuscript. All authors contributed to the article and approved the submitted version.

## Conflict of Interest

The authors declare that the research was conducted in the absence of any commercial or financial relationships that could be construed as a potential conflict of interest.

## Publisher’s Note

All claims expressed in this article are solely those of the authors and do not necessarily represent those of their affiliated organizations, or those of the publisher, the editors and the reviewers. Any product that may be evaluated in this article, or claim that may be made by its manufacturer, is not guaranteed or endorsed by the publisher.

## Supplementary Material

Supplementary Figure S1 Tumor microenvironment analysis in high and low risk groups in different subgroups. **(A)** Differences in 22 immune cells between the high and low groups in alcohol-related HCC. **(B)** Differences in 22 immune cells between the high and low groups in age <60 subgroups. **(C)** Differences in 22 immune cells between the high and low groups in staging I/II subgroups.

Supplementary Figure S2 Comparison of tumor purity between high and low risk groups and correlation analysis between two genes and 22 immune cells. **(A)** Comparison of stromal score between high and low risk groups. **(B)** Comparison of immune score between high and low risk groups. **(C)** Spearman correlation analysis between two genes and 22 immune cells. *P < 0.05, **P < 0.01.
