# Supplementary material for: Identification of a Prognostic Model Based on 2-Gene Signature and Analysis of Corresponding Tumor Microenvironment in Alcohol-Related Hepatocellular Carcinoma
Source: Front Oncol. 2021 Sep 27;11:719355. doi: 10.3389/fonc.2021.719355 (PMC8503534; doi:10.3389/fonc.2021.719355)
Supplement: Supplementary Figure S1 — Tumor microenvironment analysis in high and low risk groups in different subgroups. (A) Differences in 22 immune cells between the high and low groups in alcohol-related HCC. (B) Differences in 22 immune cells between the high and low groups in age <60 subgroups. (C) Differences in 22 immune cells between the high and low groups in staging I/II subgroups. [file DataSheet_1.docx]

**Supplementary materials**

**Table S1.** PubMed search strategy.

**Table S2.** The Newcastle-Ottawa scale (NOS) quality assessment of the enrolled studies.

**Table S3.** Summary of study characteristics, safety and efficacy of all included case report and case series.

**Table S4.** Incidence of recurrent immune-related adverse event in different ICIs retreatment.

**Table S5.** Incidence of recurrent immune-related adverse event in different initial and restart ICIs treatment.

**Table S6.** Factors associated with ORR or DCR after ICIs rechallenge.

**Figure S1.** Sensitivity analysis of the impact of each individual study on the pooled effect. A) all-grade irAEs; B) high-grade irAEs; C) ORR; D) DCR.

**Table S1. PubMed search strategy.**

| **# No** | **Searches** |
| --- | --- |
| Part I: Immune checkpoint inhibitor | |
| **1** | “Programmed Cell Death 1 Receptor”[Mesh] OR “CTLA-4 Antigen”[Mesh] |
| **2** | “anti-PD-1”[Title/Abstract] OR “PD-1”[Title/Abstract] OR “anti-PD-L1”[Title/Abstract] OR “PD-L1”[Title/Abstract] OR “anti-PD(L)-1”[Title/Abstract] OR “PD(L)-1”[Title/Abstract] OR “CTLA-4”[Title/Abstract] OR “anti-CTLA-4”[Title/Abstract] OR ” anti-cytotoxic T-lymphocyte antigen-4”[Title/Abstract] |
| **3** | “nivolumab”[Title/Abstract] OR “pembrolizumab”[Title/Abstract] OR “atezolizumab”[Title/Abstract] OR “durvalumab”[Title/Abstract] OR “avelumab”[Title/Abstract] OR “ipilimumab”[Title/Abstract] OR “cemiplimab”[Title/Abstract] |
| **4** | “immune checkpoint inhibitor”[Title/Abstract] OR “immune checkpoint inhibitors”[Title/Abstract] OR “ICI”[Title/Abstract] OR “immune checkpoint blockade”[Title/Abstract] OR “ICB”[Title/Abstract] |
| **5** | 1 or 2 or 3 or 4 |
| Part II: Cancer | |
| **6** | “cancer”[Title/Abstract] OR “tumor”[Title/Abstract] OR “tumour”[Title/Abstract] OR “neoplasm”[Title/Abstract] OR “carcinoma”[Title/Abstract] |
| Part III: Rechallenge | |
| **7** | readministrat*[Title/Abstract] OR re-administrat*[Title/Abstract] OR rechalleng*[Title/Abstract] OR re-challeng*[Title/Abstract] OR resum*[Title/Abstract] OR restart*[Title/Abstract] OR re-start*[Title/Abstract] OR retreat*[Title/Abstract] OR re-treat*[Title/Abstract] OR re-initiate[Title/Abstract] |
| Part IV: Part I, II, AND III | |
| **8** | 5 and 6 and 7 |

**Table S2. The Newcastle-Ottawa scale (NOS) quality assessment of the enrolled studies.**

| **Study ID** | **SELECTION** | | | | **COMPARABILITY** | **OUTCOME** | | | **Total^a^** |
| --- | --- | --- | --- | --- | --- | --- | --- | --- | --- |
|  | **Representativeness of the exposed cohort** | **Selection of the nonexposed cohort** | **Ascertainment of exposure** | **Demonstration that outcome of interest was not present at start of study^b^** | **Comparability of cohorts on the basis of the design or analysis^c^** | **Assessment of outcome** | **Was follow-up long enough for outcomes to occur^d^** | **Adequacy of follow up of cohorts^e^** |  |
| Abu-Sbeih 2019 | somewhat* | same institute* | record* | no | - | record* | no | not clear | 4 |
| Abu-Sbeih 2018 | somewhat* | same institute* | record* | no | - | record* | no | not clear | 4 |
| Amode 2016 | truly* | same institute* | record* | yes* | * | record* | no | not clear | 6 |
| Cortazar 2020 | truly* | same institute* | record* | no | * | record* | no | not clear | 5 |
| Koyauchi 2020 | truly* | same institute* | record* | no | * | record* | yes* | yes* | 7 |
| Menzies 2017 | truly* | same institute* | record* | no | - | record* | yes* | yes* | 6 |
| Morse 2019 | truly* | same institute* | record* | no | * | record* | no | yes* | 6 |
| Miller 2019 | truly* | same institute* | record* | no | * | record* | no | not clear | 5 |
| Mouri 2019 | truly* | same institute* | record* | no | * | record* | no | not clear | 5 |
| Naidoo 2017 | truly* | same institute* | record* | no | * | record* | no | not clear | 5 |
| Nomura 2017 | somewhat* | same institute* | record* | no | * | record* | yes* | yes* | 7 |
| Pollack 2018 | truly* | same institute* | record* | no | * | record* | no | not clear | 5 |
| Santini 2018 | truly* | same institute* | record* | no | - | record* | yes* | yes* | 6 |
| Williams 2017 | somewhat* | same institute* | record* | no | - | record* | no | not clear | 4 |
| Fujita 2019 | truly* | same institute* | record* | no | * | record* | yes* | not clear | 6 |
| Fujita 2018 | truly* | same institute* | record* | no | * | record* | no | not clear | 5 |
| Delyon 2019 | somewhat* | same institute* | record* | no | - | record* | no | not clear | 4 |
| Dubey 2020 | truly* | same institute* | record* | no | * | record* | yes* | not clear | 6 |

- indicates Zero score, * indicates one score, ** indicates two scores

^a^ Each study could be awarded a maximum of nine stars: a maximum of two stars for the item regarding comparability and a maximum of one star for the other 7 items;

^b^ One score was awarded if a study was a prospective cohort study;

^c^ A maximum of two stars could be awarded for this item. If a study performed landmark analysis, one score was awarded. If a study adjusted for confounding factors (eg. ECOG PS, age, metastases status, serum low density lipoprotein level, prior treatment line, etc.), an additional score was awarded;

^d^ For studies reporting OS or PFS, if median OS or PFS was reached, one score was awarded. For studies reporting both OS and PFS, only if median OS and PFS were both reached, one score was awarded;

^e^ If a study reported a follow-up rate of more than or equal to 80%, one score was awarded.

**Table S3. Summary of study characteristics, safety and efficacy of all included case report and case series.**

| **Study** | **Sex** | **Age** | **Cancer type** | **Initial ICIs types** | **Initial irAEs** | | **Corticosteroid dosage^a^** | **Timing for initial irAEs to ICIs rechallenge (wk)** | **Rechallenged ICIs types** | **irAEs recurrence** | **Rechallenged irAEs** | | **Tumor response after rechallenge** |
| --- | --- | --- | --- | --- | --- | --- | --- | --- | --- | --- | --- | --- | --- |
|  |  |  |  |  | **Type** | **Grade** |  |  |  |  | **Type** | **Grade** |  |
| Bat (2019) | Female | 86 | other | PD-1/PD-L1 | dermatologic | 3 | High dose | 72 | PD-1/PD-L1 | No | NA | NA | PR |
| Donato (2019) | Male | 63 | lung cancer | PD-1/PD-L1 | respiratory | 2 | NA | 3 | PD-1/PD-L1 | Yes | respiratory | 3 | PR |
| Gauci (2017) | Male | 73 | melanoma | PD-1/PD-L1 | endocrine | 3 | NA | 3.5 | PD-1/PD-L1 | No | NA | NA | PR |
| Theillac (2017) | Male | 55 | melanoma | PD-1/PD-L1 | ocular | 3 | High dose | 4 | PD-1/PD-L1 | No | NA | NA | PD |
| Samra (2016) | Male | 82 | melanoma | PD-1/PD-L1 | ocular | 3 | Moderate dose | 6 | PD-1/PD-L1 | Yes | ocular | 2 | NA |
| Lee (2020) | Female | 37 | lung cancer | PD-1/PD-L1 | rheumatologic | 3 | High dose | 8 | PD-1/PD-L1 | No | NA | NA | PD |
| Patil (2019) | Male | 52 | other | PD-1/PD-L1 | dermatologic | 3 | High dose | 3 | PD-1/PD-L1 | No | NA | NA | PD |
| Sise (2019) | Male | 60 | melanoma | CTLA-4 | gastrointestinal | 3 | High dose | 24 | PD-1/PD-L1 | Yes | nephritic | 3 | SD |
| Spain (2017) | Male | 68 | melanoma | Combination | neurologic | 3 | High dose | 40 | Combination | Yes | gastrointestinal | 3 | SD |
| Spain (2017) | Female | 62 | melanoma | Combination | gastrointestinal | NA | High dose | 96 | Combination | Yes | gastrointestinal | 3 | SD |
| Spain (2017) | Female | 39 | melanoma | Combination | gastrointestinal | 3 | High dose | 40 | Combination | No | NA | NA | SD |
| Andrea (2020) | Female | 50 | other | Combination | respiratory | 2 | High dose | 14 | PD-1/PD-L1 | Yes | gastrointestinal | 3 | PR |
| Munakata (2017) | Male | 72 | hematologic | PD-1/PD-L1 | endocrine | 3 | NA | NA | PD-1/PD-L1 | No | NA | NA | NA |
| Tay (2018) | Male | 74 | lung cancer | PD-1/PD-L1 | hematologic | 3 | High dose | 9 | PD-1/PD-L1 | Yes | hematologic | 4 | SD |
| Delyon (2019) | Male | 87 | other | PD-1/PD-L1 | rheumatologic | 2 | Moderate dose | 22 | PD-1/PD-L1 | No | NA | NA | PR |
| Delyon (2019) | Male | 61 | melanoma | Combination | rheumatologic | 1 | High dose | 29 | PD-1/PD-L1 | No | NA | NA | PD |
| Assi (2013) | Male | 54 | melanoma | CTLA-4 | endocrine | 2 | High dose | 20 | CTLA-4 | No | NA | NA | SD |
| Romain (2016) | Female | 54 | melanoma | CTLA-4 | gastrointestinal | 1 | Moderate dose | 10 | CTLA-4 | No | NA | NA | NA |
| Kitchlu (2017) | Male | 45 | melanoma | CTLA-4 | renal | 3 | High dose | 104 | CTLA-4 | Yes | nephritic | 3 | PD |
| Ludlow (2016) | Male | 53 | melanoma | CTLA-4 | gastrointestinal | 3 | High dose | NA | PD-1/PD-L1 | Yes | rheumatologic | 3 | PD |
| Khan (2017) | Female | 53 | lung cancer | PD-1/PD-L1 | endocrine | 3 | NA | NA | PD-1/PD-L1 | No | NA | NA | NA |
| Mishima (2019) | Male | 63 | lung cancer | PD-1/PD-L1 | endocrine | 3 | High dose | 5 | PD-1/PD-L1 | No | NA | NA | NA |
| Obara (2017) | Female | 74 | lung cancer | PD-1/PD-L1 | dermatologic | 2 | High dose | 36 | PD-1/PD-L1 | No | NA | NA | NA |
| Nishino (2018) | Female | 50 | hematologic | PD-1/PD-L1 | respiratory | 2 | High dose | 5 | PD-1/PD-L1 | No | NA | NA | NA |
| Nishino (2018) | Female | 72 | hematologic | PD-1/PD-L1 | respiratory | 2 | High dose | 5 | PD-1/PD-L1 | No | NA | NA | NA |
| Nishino (2018) | Male | 53 | hematologic | PD-1/PD-L1 | respiratory | 2 | High dose | 5 | PD-1/PD-L1 | No | NA | NA | NA |
| Nishino (2018) | Female | 62 | hematologic | PD-1/PD-L1 | respiratory | 2 | High dose | 5 | PD-1/PD-L1 | Yes | respiratory | NA | NA |
| Matsuoka (2020) | Male | 57 | lung cancer | PD-1/PD-L1 | endocrine | 3 | NA | NA | PD-1/PD-L1 | No | NA | NA | NA |
| Anastasia (2019) | Male | 65 | lung cancer | PD-1/PD-L1 | respiratory | 4 | NA | 64 | PD-1/PD-L1 | No | NA | NA | NA |
| Kato (2017) | Female | 39 | lung cancer | PD-1/PD-L1 | respiratory | 2 | High dose | 12 | PD-1/PD-L1 | Yes | respiratory | 2 | PR |
| Brilli (2020) | Male | 68 | other | Combination | endocrine | 2 | NA | 4 | Combination | No | NA | NA | PD |
| Carausu (2019) | Male | 68 | lung cancer | PD-1/PD-L1 | neurologic | 3 | High dose | 14 | PD-1/PD-L1 | No | NA | NA | PR |
| Saito (2020) | Male | 79 | lung cancer | PD-1/PD-L1 | renal | 3 | High dose | NA | PD-1/PD-L1 | No | NA | NA | SD |
| Fujii (2018) | Female | 82 | melanoma | PD-1/PD-L1 | gastrointestinal | 3 | High dose | 14 | PD-1/PD-L1 | No | NA | NA | SD |
| Raskin (2017) | Male | 58 | lung cancer | PD-1/PD-L1 | neurologic | 2 | High dose | 5 | PD-1/PD-L1 | Yes | neurologic | 2 | NA |
| Hu (2020) | Male | 66 | other | PD-1/PD-L1 | renal | 3 | Moderate dose | 4 | PD-1/PD-L1 | No | NA | NA | NA |
| Gauci (2017) | Male | 80 | melanoma | PD-1/PD-L1 | rheumatologic | 3 | Moderate dose | 4 | PD-1/PD-L1 | Yes | rheumatologic | 1 | PD |
| De Martin (2019) | Female | 64 | melanoma | Combination | gastrointestinal | NA | High dose | NA | PD-1/PD-L1 | Yes | gastrointestinal | NA | NA |
| Iyoda (2018) | Male | 62 | lung cancer | PD-1/PD-L1 | gastrointestinal | 3 | High dose | NA | PD-1/PD-L1 | Yes | gastrointestinal | 3 | SD |
| Gravbrot (2019) | Male | 71 | melanoma | CTLA-4 | neurologic | 3 | Moderate dose | 54 | PD-1/PD-L1 | No | NA | NA | CR |
| Frohlich (2020) | Male | 57 | melanoma | CTLA-4 | rheumatologic | NA | Moderate dose | NA | CTLA-4 | Yes | endocrine | 1 | CR |
| Frohlich (2020) | Male | 53 | melanoma | PD-1/PD-L1 | rheumatologic | NA | NA | NA | PD-1/PD-L1 | No | NA | NA | PD |
| Reddy (2017) | Female | 55 | melanoma | Combination | rheumatologic | NA | High dose | 10 | PD-1/PD-L1 | No | NA | NA | SD |
| Reddy (2017) | Female | 57 | melanoma | PD-1/PD-L1 | gastrointestinal | 4 | High dose | 20 | PD-1/PD-L1 | No | NA | NA | PR |
| Gao (2019) | Male | 60 | melanoma | Combination | neurologic | NA | High dose | 16 | PD-1/PD-L1 | No | NA | NA | PD |
| Mancone (2018) | Male | 62 | renal | PD-1/PD-L1 | neurologic | 2 | NA | <1 | PD-1/PD-L1 | Yes | neurologic | 2 | NA |
| Mancone (2018) | Male | 57 | melanoma | Combination | neurologic | 4 | NA | 4 | PD-1/PD-L1 | Yes | neurologic | 2 | PD |
| Dasanu (2020) | Male | 58 | other | PD-1/PD-L1 | gastrointestinal | 3 | High dose | 4 | PD-1/PD-L1 | No | NA | NA | PR |
| Kobayashi (2020) | Male | 69 | lung cancer | PD-1/PD-L1 | gastrointestinal | 4 | High dose | 5 | PD-1/PD-L1 | No | NA | NA | CR |
| Shiuan (2017) | Female | 47 | melanoma | Combination | hematologic | 3 | NA | 1 | PD-1/PD-L1 | No | NA | NA | PR |
| Shiuan (2017) | Female | 45 | melanoma | PD-1/PD-L1 | hematologic | 3 | NA | 15 | CTLA-4 | Yes | hematologic | 3 | NA |
| Muralikrishnan (2020) | Female | 65 | melanoma | PD-1/PD-L1 | neurologic | 3 | High dose | 52 | CTLA-4 | Yes | gastrointestinal | 1 | PR |
| Azmat (2016) | Male | 67 | melanoma | CTLA-4 | endocrine | 2 | NA | NA | CTLA-4 | No | NA | NA | NA |
| Imafuku (2017) | Male | 62 | melanoma | PD-1/PD-L1 | respiratory | 1 | Low dose | NA | PD-1/PD-L1 | No | NA | NA | NA |
| Imafuku (2017) | Male | 75 | melanoma | PD-1/PD-L1 | respiratory | 2 | High dose | NA | PD-1/PD-L1 | Yes | respiratory | 2 | NA |
| Telfah (2019) | Male | 57 | melanoma | PD-1/PD-L1 | ocular | 2 | NA | 16 | PD-1/PD-L1 | Yes | neurologic | NA | NA |
| Kosche (2019) | Female | 75 | other | Combination | rheumatologic | 2 | High dose | 2 | Combination | Yes | rheumatologic | 2 | NA |
| Khan (2017) | Female | 43 | melanoma | Combination | hematologic | 3 | High dose | 8 | Combination | Yes | hematologic | / | SD |
| Leaf (2019) | Female | 67 | melanoma | Combination | hematologic | 2 | High dose | NA | PD-1/PD-L1 | No | NA | NA | CR |
| Leaf (2019) | Male | 68 | melanoma | PD-1/PD-L1 | hematologic | NA | High dose | NA | PD-1/PD-L1 | No | NA | NA | CR |
| Leaf (2019) | Male | 67 | melanoma | Combination | hematologic | NA | High dose | NA | Combination | No | NA | NA | SD |
| Leaf (2019) | Male | 55 | melanoma | PD-1/PD-L1 | hematologic | NA | High dose | NA | PD-1/PD-L1 | No | NA | NA | SD |
| Baseri (2019) | Male | 73 | lung cancer | PD-1/PD-L1 | dermatologic | 2 | High dose | 92 | PD-1/PD-L1 | No | NA | NA | PR |
| Dizman (2018) | Male | 44 | renal | Combination | gastrointestinal | 3 | NA | 180 | PD-1/PD-L1 | Yes | gastrointestinal | 3 | CR |
| Lomax (2018) | Female | 78 | melanoma | PD-1/PD-L1 | dermatologic | 4 | High dose | 24 | PD-1/PD-L1 | No | NA | NA | NA |
| Tedbirt (2019) | Male | 77 | melanoma | PD-1/PD-L1 | neurologic | NA | High dose | 44 | PD-1/PD-L1 | Yes | neurologic | NA | PR |
| Fellner (2018) | Male | 68 | melanoma | PD-1/PD-L1 | neurologic | NA | Moderate dose | NA | PD-1/PD-L1 | No | NA | NA | NA |
| Nishino (2016) | Male | 58 | melanoma | PD-1/PD-L1 | respiratory | 2 | High dose | NA | PD-1/PD-L1 | No | NA | NA | NA |
| Nishino (2016) | Male | 57 | melanoma | Combination | respiratory | 1 | NA | NA | PD-1/PD-L1 | No | NA | NA | NA |
| Nishino (2016) | Female | 66 | melanoma | Combination | respiratory | 2 | High dose | NA | PD-1/PD-L1 | Yes | respiratory | NA | NA |
| Nishino (2016) | Female | 30 | hematologic | Combination | respiratory | 2 | High dose | NA | PD-1/PD-L1 | No | NA | NA | NA |
| Nishino (2016) | Female | 33 | hematologic | Combination | respiratory | 2 | High dose | NA | PD-1/PD-L1 | Yes | respiratory | NA | NA |
| Sun (2018) | Male | 64 | melanoma | Combination | hematologic | 3 | High dose | 15.4 | PD-1/PD-L1 | NA | NA | NA | PR |
| Tardy (2017) | Female | 75 | hematologic | PD-1/PD-L1 | hematologic | 3 | High dose | 62 | PD-1/PD-L1 | NA | NA | NA | PD |
| Kitagawa (2020) | NA | NA | lung cancer | PD-1/PD-L1 | NA | NA | NA | NA | PD-1/PD-L1 | NA | NA | NA | PD |
| Kitagawa (2020) | NA | NA | lung cancer | PD-1/PD-L1 | NA | NA | NA | NA | PD-1/PD-L1 | NA | NA | NA | SD |
| Kitagawa (2020) | NA | NA | lung cancer | PD-1/PD-L1 | NA | NA | NA | NA | PD-1/PD-L1 | NA | NA | NA | SD |
| Kitagawa (2020) | NA | NA | lung cancer | PD-1/PD-L1 | NA | NA | NA | NA | PD-1/PD-L1 | NA | NA | NA | SD |
| Kitagawa (2020) | NA | NA | lung cancer | PD-1/PD-L1 | NA | NA | NA | NA | PD-1/PD-L1 | NA | NA | NA | SD |
| Kitagawa (2020) | NA | NA | lung cancer | PD-1/PD-L1 | NA | NA | NA | NA | PD-1/PD-L1 | NA | NA | NA | PD |
| Kitagawa (2020) | NA | NA | lung cancer | PD-1/PD-L1 | NA | NA | NA | NA | PD-1/PD-L1 | NA | NA | NA | SD |
| Kitagawa (2020) | NA | NA | lung cancer | PD-1/PD-L1 | NA | NA | NA | NA | PD-1/PD-L1 | NA | NA | NA | SD |
| Kitagawa (2020) | NA | NA | lung cancer | PD-1/PD-L1 | NA | NA | NA | NA | PD-1/PD-L1 | NA | NA | NA | PD |
| Kitagawa (2020) | NA | NA | lung cancer | PD-1/PD-L1 | NA | NA | NA | NA | PD-1/PD-L1 | NA | NA | NA | PD |
| Kitagawa (2020) | NA | NA | lung cancer | PD-1/PD-L1 | NA | NA | NA | NA | PD-1/PD-L1 | NA | NA | NA | PD |
| Kitagawa (2020) | NA | NA | lung cancer | PD-1/PD-L1 | NA | NA | NA | NA | PD-1/PD-L1 | NA | NA | NA | PD |
| Kitagawa (2020) | NA | NA | lung cancer | PD-1/PD-L1 | NA | NA | NA | NA | PD-1/PD-L1 | NA | NA | NA | SD |
| Kitagawa (2020) | NA | NA | lung cancer | PD-1/PD-L1 | NA | NA | NA | NA | PD-1/PD-L1 | NA | NA | NA | PD |
| Kitagawa (2020) | NA | NA | lung cancer | PD-1/PD-L1 | NA | NA | NA | NA | PD-1/PD-L1 | NA | NA | NA | PR |
| Kitagawa (2020) | NA | NA | lung cancer | PD-1/PD-L1 | NA | NA | NA | NA | PD-1/PD-L1 | NA | NA | NA | SD |
| Kitagawa (2020) | NA | NA | lung cancer | PD-1/PD-L1 | NA | NA | NA | NA | PD-1/PD-L1 | NA | NA | NA | SD |
| Niki (2018) | NA | NA | lung cancer | PD-1/PD-L1 | NA | NA | NA | NA | PD-1/PD-L1 | NA | NA | NA | PR |
| Niki (2018) | NA | NA | lung cancer | PD-1/PD-L1 | NA | NA | NA | NA | PD-1/PD-L1 | NA | NA | NA | PR |
| Niki (2018) | NA | NA | lung cancer | PD-1/PD-L1 | NA | NA | NA | NA | PD-1/PD-L1 | NA | NA | NA | PR |
| Niki (2018) | NA | NA | lung cancer | PD-1/PD-L1 | NA | NA | NA | NA | PD-1/PD-L1 | NA | NA | NA | SD |
| Niki (2018) | NA | NA | lung cancer | PD-1/PD-L1 | NA | NA | NA | NA | PD-1/PD-L1 | NA | NA | NA | SD |
| Niki (2018) | NA | NA | lung cancer | PD-1/PD-L1 | NA | NA | NA | NA | PD-1/PD-L1 | NA | NA | NA | SD |

Abbreviations: CR: complete response; CTLA-4: cytotoxic T-lymphocyte antigen-4; ICIs: immune checkpoint inhibitors; irAEs: immune-related adverse events; NA: not applicable; PD: progressive disease; PD-1: programmed cell death protein-1; PD-L1: programmed cell death protein ligand-1; PR: partial response; SD: stable disease.

^a^ Low dose was considered as “prednisone≤7.5 mg/d” or “methylprednisolone≤6 mg/d”; Moderate dose was considered as “7.5 mg/d<prednisone≤30 mg/d” or “6 mg/d<methylprednisolone≤24 mg/d”; High dose was considered as “prednisone>30 mg/d” or “methylprednisolone>24 mg/d”.

**Table S4. Incidence of recurrent immune-related adverse event in different ICIs retreatment.**

|  | **CTLA-4** | **PD-1/PD-L1** | **combination** |
| --- | --- | --- | --- |
| Abu-Sbeih | 14/32 | 43/135 | NA |
| Amode | NA | 14/23 | NA |
| Koyauchi | NA | 5/16 | NA |
| Menzies | NA | 25/67 | NA |
| Morse | NA | NA | 14/25 |
| Miller | 1/5 | 7/25 | 0/1 |
| Mouri | NA | 15/21 | NA |
| Naidoo | NA | 3/24 | 0/19 |
| Pollack | NA | 40/80 | NA |
| Santini | NA | 20/38 | NA |
| Delyon | 0/2 | 0/8 | 1/1 |
| Total | 15/39=0.385 | 172/437=0.394 | 15/46=0.326 |
| Analysis | χ^2^=0.800, p=0.670, df=2 | | |

CTLA-4: cytotoxic T-lymphocyte antigen-4; ICIs: immune checkpoint inhibitors; irAEs: immune-related adverse events; NA: not applicable; PD-1: programmed cell death protein-1; PD-L1: programmed cell death protein ligand-1.

**Table S5. Incidence of recurrent immune-related adverse event in different initial and restart ICIs treatment.**

| **Initial ICIs** | **CTLA-4** | | | **PD-1/PDL-1** | | | **Combination** | | |
| --- | --- | --- | --- | --- | --- | --- | --- | --- | --- |
| **Rechallenged ICIs** | **CTLA-4** | **PD-1/PDL-1** | **combination** | **CTLA-4** | **PD-1/PDL-1** | **combination** | **CTLA-4** | **PD-1/PDL-1** | **combination** |
| Abu-Sbeih1 | 7/24 | 17/64 | NA | 7/8 | 26/71 | NA | NA | NA | NA |
| Amode | NA | 14/23 | NA | NA | NA | NA | NA | NA | NA |
| Koyauchi | NA | NA | NA | NA | 5/16 | NA | NA | NA | NA |
| Menzies | NA | NA | NA | NA | 25/67 | NA | NA | NA | NA |
| Morse | NA | NA | NA | NA | NA | NA | NA | NA | 14/25 |
| Miller | 1/3 | 1/5 | NA | 0/1 | 3/12 | NA | 0/1 | 3/8 | 0/1 |
| Mouri | NA | NA | NA | NA | 15/21 | NA | NA | NA | NA |
| Pollack | NA | NA | NA | NA | NA | NA | NA | 40/80 | NA |
| Santini | NA | NA | NA | NA | 13/24 | NA | NA | 7/14 | NA |
| Total | 8/27=0.296 | NA |  | 7/9=0.778 | 87/211=0.412 | NA | 0 | 50/102=0.490 | 14/25=0.560 |
| Analysis | χ^2^=0.248, p=0.618, df=1 | | NA | p=0.040^a^ | | NA | NA | χ^2^=0.391, p=0.532, df=1 | |

CTLA-4: cytotoxic T-lymphocyte antigen-4; ICIs: immune checkpoint inhibitors; NA: not applicable; PD-1: programmed cell death protein-1; PD-L1: programmed cell death protein ligand-1.

^a^ Fisher exact test was performed.

**Table S6. Factors associated with ORR or DCR after ICIs rechallenge**

|  | **ORR** | | | | **DCR** | | | |
| --- | --- | --- | --- | --- | --- | --- | --- | --- |
|  | **n (total numbers, %)^a^** | | **Univariate analysis** | | **n (total numbers, %)^a^** | | **Univariate analysis** | |
|  | **Recurrence** | **Non- Recurrence** | **OR (95%CI)** | ***p*** | **Recurrence** | **Non- Recurrence** | **OR (95%CI)** | ***p*** |
| **Age** | 64.15±12.78 | 59.84±12.28 | 1.03(0.98,1.08) | 0.253 | 63.12±12.63 | 58.00±12.03 | 1.04(0.98,1.09) | 0.228 |
| **Gender (Male)** | 13(20,65.0%) | 18(25,72.0%) | 1.39(0.39,4.92) | 0.615 | 21(33,63.6%) | 10(12,83.3%) | 2.86(0.54,15.26) | 0.219 |
| **Types of initial irAEs** |  |  |  |  |  |  |  |  |
| Gastrointestinal | 4(20,20.0%) | 6(25,24.0%) | 0.79(0.19,3.31) | 0.749 | 9(33,27.3%) | 1(12,8.3%) | 4.13(0.46,36.70) | 0.204 |
| Nephritic | 0(20,0.0%) | 2(25,8.0%) | NA | 0.999 | 1(33,3.0%) | 1(12,8.3%) | 0.34(0.02,5.97) | 0.464 |
| Hematologic | 4(20,20.0%) | 5(25,20.0%) | 1.00(0.23,4.35) | 1.000 | 8(33,24.2%) | 1(12,8.3%) | 3.52(0.39,31.66) | 0.261 |
| Neurologic | 4(20,20.0%) | 3(25,12.0%) | 1.83(0.36,9.35) | 0.466 | 5(33,15.2%) | 2(12,16.7%) | 0.89(0.15,5.36) | 0.901 |
| Endocrine | 1(20,5.0%) | 2(25,8.0%) | 0.61(0.05,7.20) | 0.691 | 2(33,6.1%) | 1(12,8.3%) | 0.71(0.06,8.62) | 0.788 |
| Respiratory | 3(20,15.0%) | 0(25,0.0%) | NA | 0.999 | 3(33,9.1%) | 0(12,0.0%) | NA | 0.999 |
| Ocular | 0(20,0.0%) | 1(25,4.0%) | NA | 1.000 | 0(33,0.0%) | 1(12,8.3%) | NA | 1.000 |
| Rheumatologic | 2(20,10.0%) | 5(25,20%) | 0.44(0.08,2.58) | 0.366 | 3(33,9.1%) | 4(12,33.3%) | 0.20(0.04,1.08) | 0.062 |
| Dermatologic | 2(20,10.0%) | 1(25,4.0%) | 2.67(0.22,31.75) | 0.438 | 2(33,6.1%) | 1(12,8.3%) | 0.71(0.06,8.62) | 0.788 |
| **Initial irAEs grade^b^** |  |  | 0.34(0.07,1.68) | 0.186 |  |  | 0.68(0.12,4.01) | 0.669 |
| Low-grade | 6(17,35.3%) | 3(19,15.8%) |  |  | 7(26,26.9%) | 2(10,20.0%) |  |  |
| High-grade | 11(17,64.7%) | 16(19,84.2%) |  |  | 19(26,73.1%) | 8(10,80.0%) |  |  |
| **Initial corticosteroid dosage^c^** |  |  | 0.21(0.02,2.20) | 0.191 |  |  | 1.08(0.10,11.92) | 0.948 |
| Low/moderate dose | 3(16,18.8%) | 1(22,4.5%) |  |  | 3(29,10.3%) | 1(9,11.1%) |  |  |
| High dose | 13(16,81.3%) | 21(22,95.5%) |  |  | 26(29,89.7%) | 8(9,88.9%) |  |  |
| **Cancer type** |  |  |  |  |  |  |  |  |
| Melanoma | 10(24,41.7%) | 18(44,40.9%) | 1.03(0.38,2.83) | 0.952 | 20(49,40.8%) | 8(19,42.1%) | 0.95(0.32,2.78) | 0.923 |
| Lung | 9(24,37.5%) | 23(44,52.3%) | 0.55(0.20,1.51) | 0.246 | 24(49,49.0%) | 8(19,42.1%) | 1.32(0.45,3.85) | 0.611 |
| Renal | 1(24,4.2%) | 0(44,0.0%) | NA | 1.000 | 1(49,2.0%) | 0(19,0.0%) | NA | 1.000 |
| Hematologic | 0(24,0.0%) | 1(44,2.3%) | NA | 1.000 | 0(49,0.0%) | 1(19,5.3%) | NA | 1.000 |
| **Initial ICIs types** |  |  |  |  |  |  |  |  |
| PD-1/PD-L1 | 17(24,70.8%） | 30(44,68.2%） | 1.13(0.38,3.35) | 0.821 | 34(49,69.4%) | 13(19,68.4%) | 1.05(0.33,3.28) | 0.938 |
| CTLA-4 | 2(24,8.3%) | 4(44,9.1%) | 0.91(0.15,5.37) | 0.916 | 4(49,8.2%) | 2(19,10.5%) | 0.76(0.13,4.51) | 0.758 |
| Combination | 5(24,20.8%) | 10(44,22.7%) | 0.90(0.27,3.01) | 0.857 | 11(49,22.4%) | 4(19,21.1%) | 1.09(0.30,3.95) | 0.901 |
| **Time interval between initial irAEs and ICIs rechallenge (wk)** | 15.40(4.50,53.00) | 14.00(4.00,40.00) | 1.01(0.99,1.03) | 0.459 | 17.70(8.75,46.00) | 6.00(4.00,37.25) | 1.01(0.99,1.03) | 0.501 |
| **Rechallenged ICIs types** |  |  |  |  |  |  |  |  |
| PD-1/PD-L1 | 22(24,91.7%) | 36(44,81.8%) | 2.44(0.48,12.57) | 0.285 | 41(49,83.7%) | 17(19,89.5%) | 0.60(0.12,3.14) | 0.548 |
| CTLA-4 | 2(24,8.3%) | 2(44,4.5%) | 1.91(0.25,14.49) | 0.532 | 3(49,6.1%) | 1(19,5.3%) | 1.17(0.11,12.04) | 0.893 |
| Combination | 0(24,0.0%) | 6(44,13.6%) | NA | 0.999 | 5(49,10.2%) | 1(19,5.3%) | 2.05(0.22,18.76) | 0.527 |

Abbreviations: CI: confidence interval; CTLA-4: cytotoxic T-lymphocyte antigen-4; DCR: disease control rate; ICIs: immune checkpoint inhibitors; irAEs: immune-related adverse events; NA: not applicable; OR: odds ratio; ORR: objective response rate; PD-1: programmed cell death protein-1; PD-L1: programmed cell death protein ligand-1;

^a^ Qualitative variables were reported as n (total numbers, %), and quantitative variables were reported as mean ± standard deviation (SD) or a median with interquartile range (IQR);

^b^ Initial irAEs grade: Low-grade was considered as grade 1-2, and high-grade was considered as grade≥3;

^c^ Low-dose was considered as “prednisone≤7.5 mg/d” or “methylprednisolone≤6 mg/d”; Moderate-dose was considered as “7.5 mg/d<prednisone≤30 mg/d” or “6 mg/d<methylprednisolone≤24 mg/d”; High-dose was considered as “prednisone>30 mg/d” or “methylprednisolone>24 mg/d”.


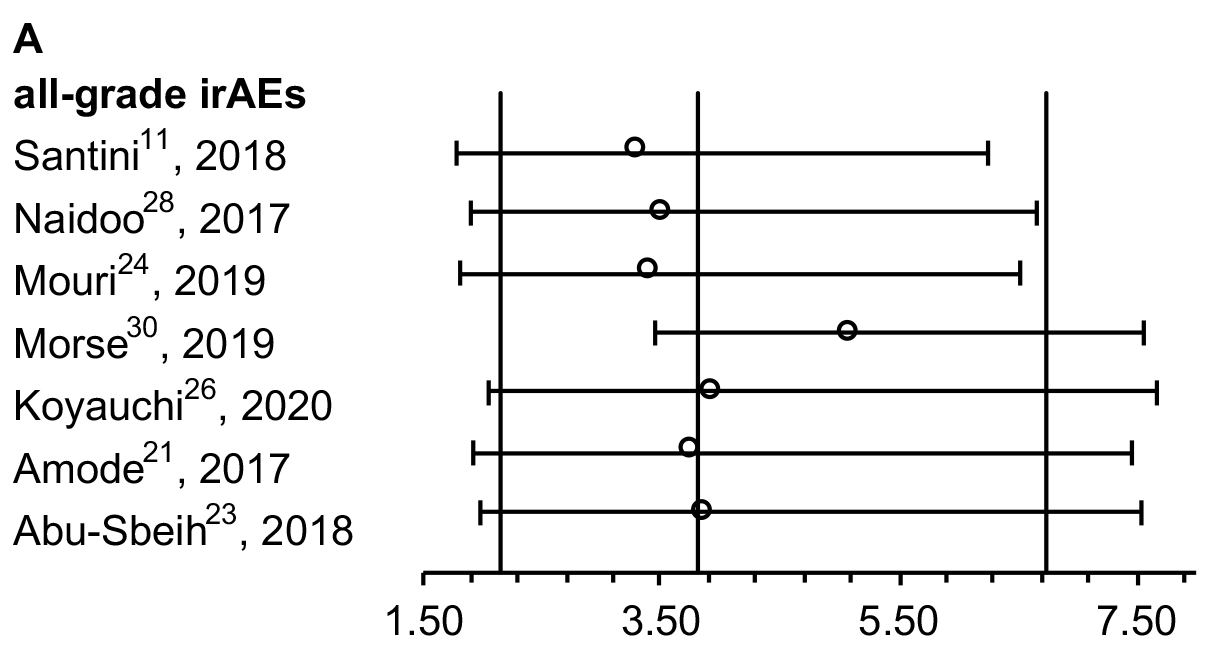


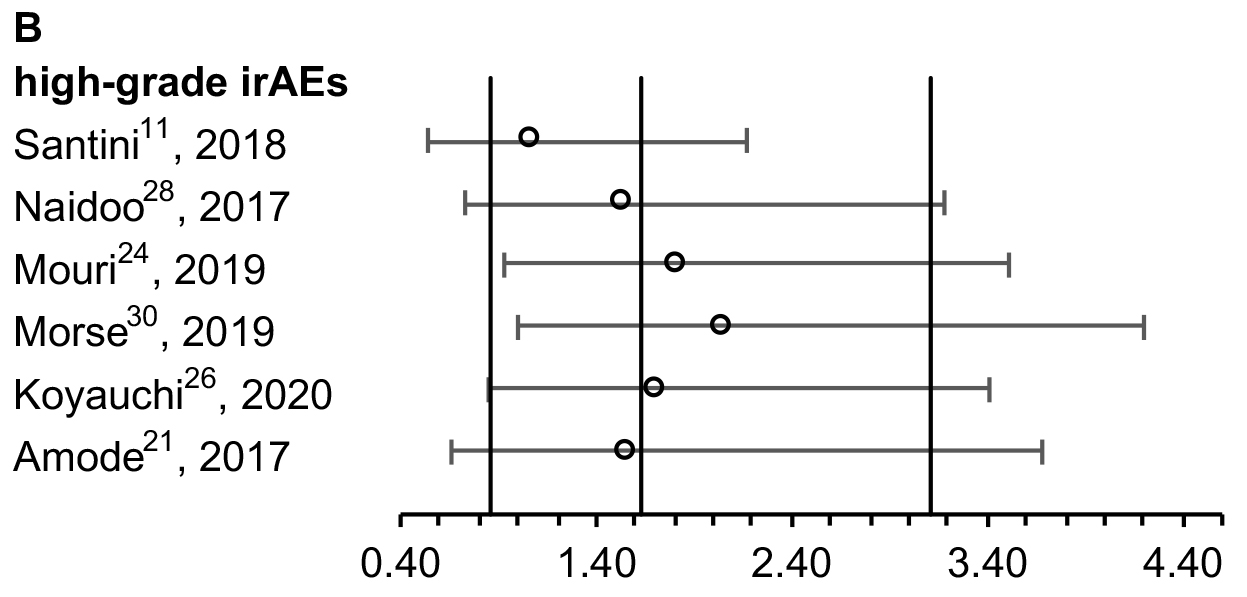


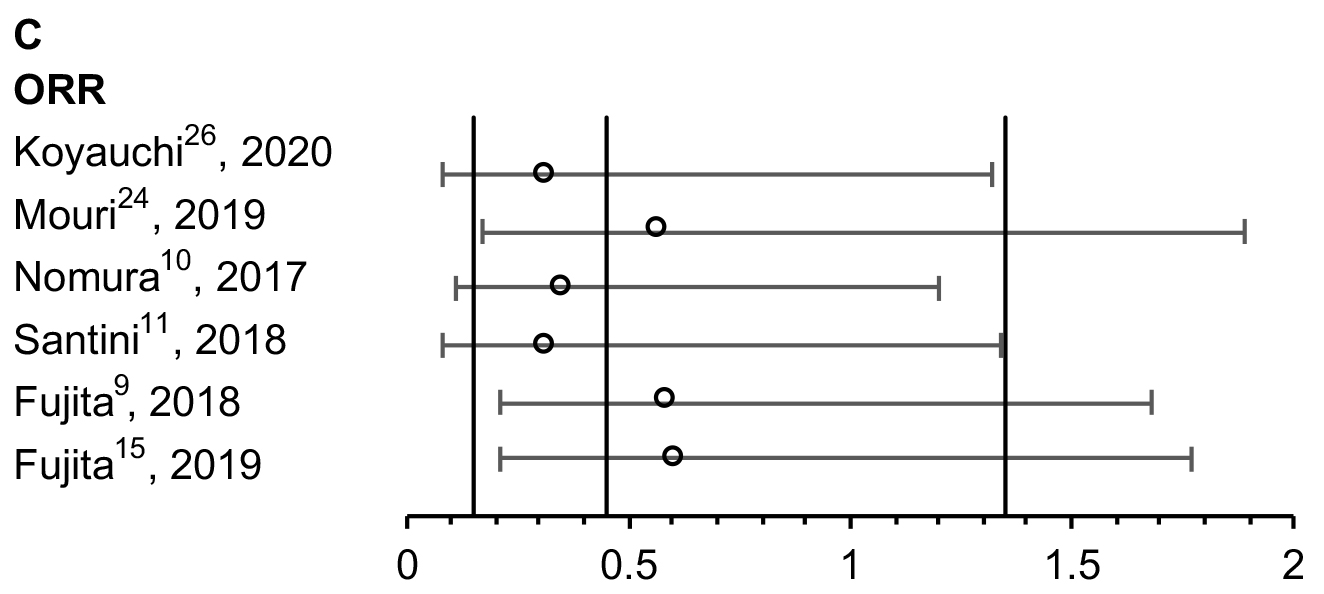


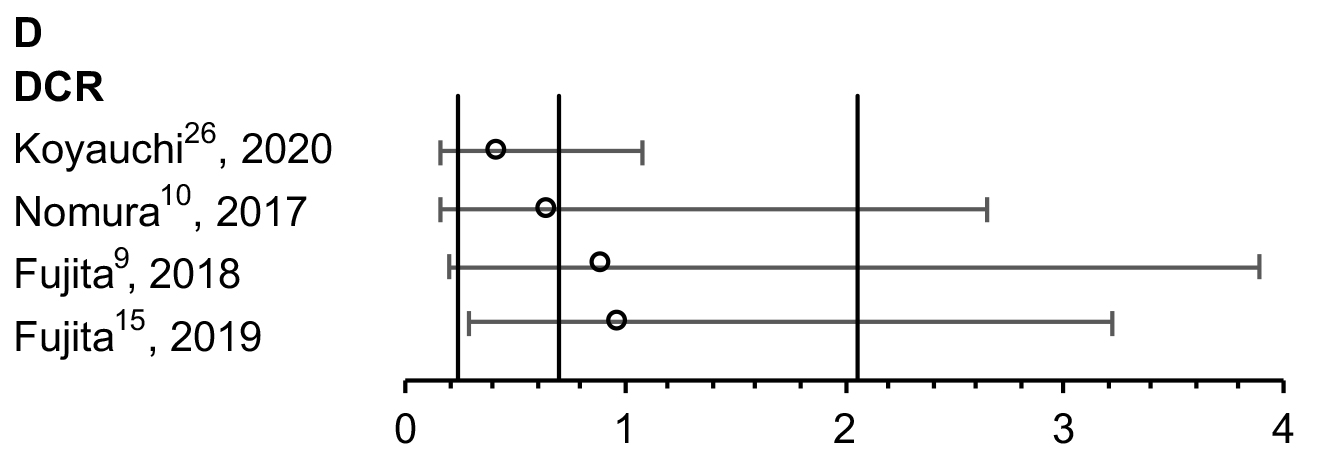


**Figure S1. Sensitivity analysis of the impact of each individual study on the pooled effect. A) all-grade irAEs; B) high-grade irAEs; C) ORR; D) DCR.**

DCR: disease control rate; irAEs: immune-related adverse event; ORR: objective response rate.

High-grade irAEs was considered as grade ≥ 3;
