# Supplementary figures and images for: Identification of a Prognostic Model Based on 2-Gene Signature and Analysis of Corresponding Tumor Microenvironment in Alcohol-Related Hepatocellular Carcinoma
Source: Front Oncol. 2021 Sep 27;11:719355. doi: 10.3389/fonc.2021.719355 (PMC8503534; doi:10.3389/fonc.2021.719355)

C

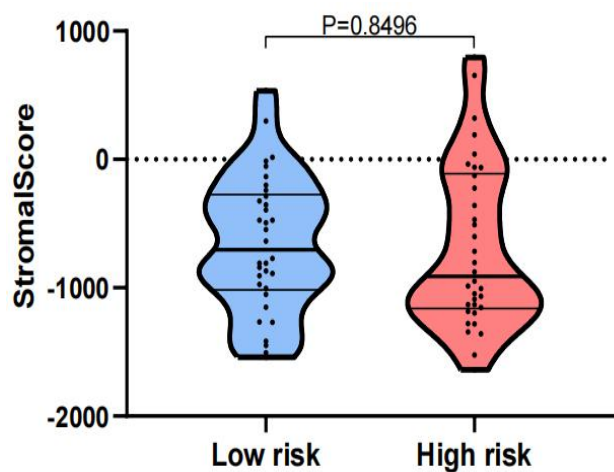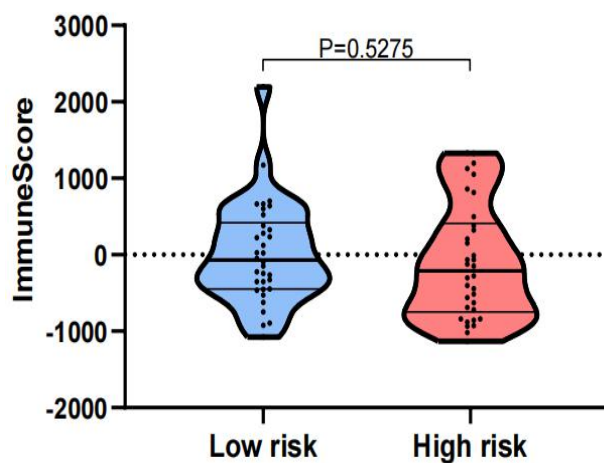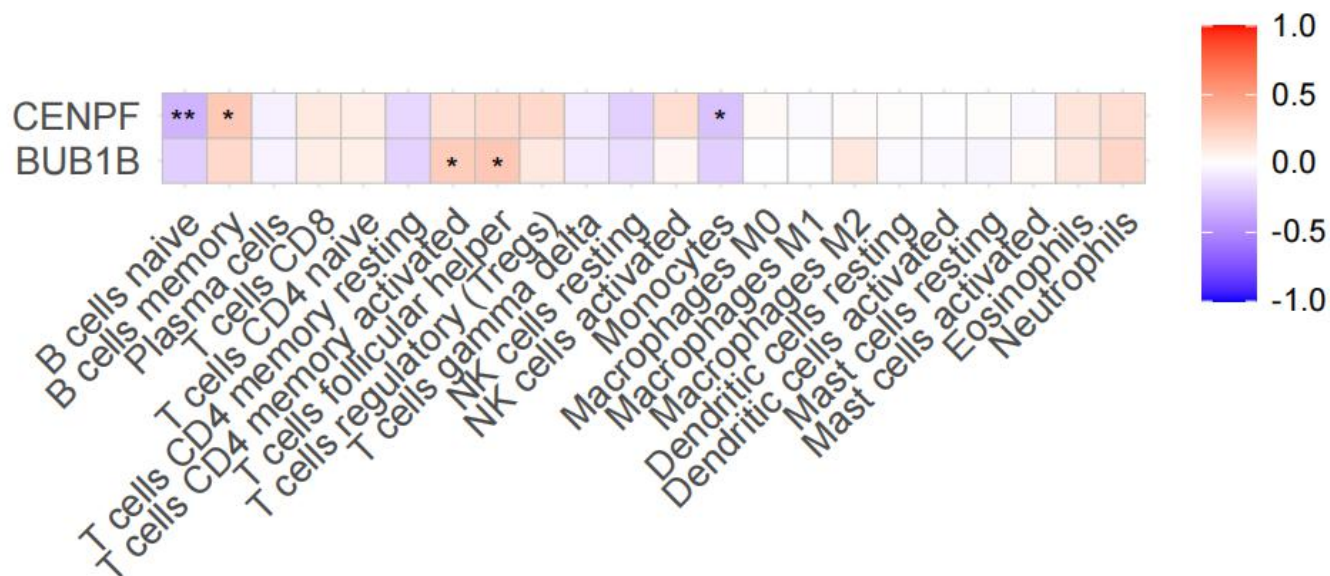

Supplement: Supplementary Figure S2 — Comparison of tumor purity between high and low risk groups and correlation analysis between two genes and 22 immune cells. (A) Comparison of stromal score between high and low risk groups. (B) Comparison of immune score between high and low risk groups. (C) Spearman correlation analysis between two genes and 22 immune cells. *P < 0.05, **P < 0.01. [file DataSheet_2.pdf]
